# Normal and Tumour Tissue mRNA Expressions of Telomerase Complex Genes in Several Types of Cancer

**DOI:** 10.4274/balkanmedj.2015.1616

**Published:** 2017-05-15

**Authors:** Emel Çalışkan Can, M. Can Atalay, Ece Miser Salihoğlu, Ülkü Yalçıntaş Arslan, H. Bolkan Şimşek, Sevgi Yardım Akaydın

**Affiliations:** 1 Department of Biochemistry, Gazi University School of Pharmacy, Ankara, Turkey; 2 Department of General Surgery, Ankara Oncology Training and Research Hospital, Ankara, Turkey; 3 Department of Medical Oncology, Ankara Oncology Training and Research Hospital, Ankara, Turkey

**Keywords:** Human telomerase reverse transcriptase, pontin, reptin, dyskerin, telomerase, Cancer

## Abstract

**Aims:**

To investigate the changes in mRNA expression levels of telomerase-related significant proteins in several types of cancer.

**Methods:**

Human telomerase reverse transcriptase, pontin, reptin and dyskerin expressions were measured in normal and tumour tissues obtained from 26 patients with colorectal, breast and gastric cancers, using the real-time reverse transcriptase-polymerase chain reaction method.

**Results:**

For all patients, no significant difference was found in mRNA expressions of human telomerase reverse transcriptase and dyskerin (p>0.05), although their levels in tumour tissues were found to be higher than in normal tissues. However, pontin and reptin mRNA expressions were significantly higher in tumour tissues than in normal tissues (p<0.01). While human telomerase reverse transcriptase showed a high correlation with only pontin (p<0.001) in normal tissues, high positive correlations were observed between human telomerase reverse transcriptase with pontin (p<0.005), reptin (p<0.01) and dyskerin (p<0.01) in tumour tissues.

**Conclusion:**

The increased mRNA expressions of all four genes in tumour tissues may suggest a role in cancer development. Correlations of pontin, reptin and dyskerin with human telomerase reverse transcriptase support the hypotheses describing their roles in telomerase complexes.

Telomerase enzyme activity is required for immortality of tumour cells and the carcinogenesis process. In human cancers, telomerase is upregulated where it serves tumour proliferation by helping in the protection of functional telomeres (1). The telomerase complex is composed of telomerase reverse transcriptase (TERT), telomerase RNA component (TERC) and dyskerin (2). TERT is the rate-limiting factor of telomerase activity (3), whereas TERC is a small nucleolar RNA (snoRNA), which is used as a template for prime telomere replication (4). Dyskerin is a TERC-binding protein, which is present in the complex structure to contribute to telomere stabilization and maintenance (5,6). Loss of the function of dyskerin decreases the steady-state levels of TERC, reduces the telomerase activity and causes premature telomere shortening (5). Along with TERT, TERC and dyskerin, two additional proteins, pontin and reptin, help in the formation of the telomerase complex. The interaction of pontin and reptin with TERT and dyskerin plays an important role in telomerase activity. Similarly, pontin, reptin and dyskerin are required for sustaining TERC levels, thereby suggesting that there is a functional relationship between these proteins (1).

Pontin and reptin are the helicase ATPases associated with diverse cellular activities (AAA+). Pontin and reptin are known as RuvB-like protein 1 (Ruvbl1) and Ruvbl2, respectively (7). Pontin is active in homologous recombination or in the repairing of the stalled replication fork with its homologue reptin (8). Pontin and reptin are also involved in a lot of cellular pathways such as transcription activation and repression, DNA damage response, small nucleolar ribonucleoprotein (snoRNP) assembly, cellular transformation, metastasis, apoptosis, DNA replication, mitosis, development and URI/prefoldin complexes (9).

The aim of this study was to compare the mRNA expressions of hTERT, pontin, reptin and dyskerin in normal and tumour tissues, and to examine the relationships between pontin, reptin and dyskerin with hTERT.

## MATERIALS AND METHODS

### Study population

Thirteen patients with colon cancer, 7 patients with breast cancer and 6 patients with gastric cancer were included in this study between October 2009 and February 2011. Seventeen patients were female (aged 31-74 years) and 9 were male (aged 46-68 years). All the patients had recently been diagnosed using the biopsy method and had not previously received any medical treatment. The histopathological diagnosis was adenocarcinoma in all patients whether they had colorectal, gastric or breast tumours. Tumour and non-tumour tissue samples were obtained from patients during surgical resection of the tumour. Macroscopically, tumour-free margins were obtained during surgery and non-tumour tissue samples were collected from macroscopically normal areas in the stomach, colon, rectum and breast. Patients were classified according to the TNM system, developed by the American Joint Committee on Cancer (10). Informed consent was obtained from the patients before starting the study in accordance with the Helsinki Declaration. This study was approved by the Clinical Research Ethics Committee. Basic characteristics of the patients are given in Table 1.

### Sample collection and storage

Sufficient amounts of distant non-tumour tissues of the colon, breast and stomach were obtained from the patients, as control tissues. All tumour tissue samples and distant normal tissue samples were immediately placed in microtubes filled with RNA Safer^TM^ RNA Stabilization Reagent (SABiosciences; Frederick, MA, USA) during surgical removal, frozen and stored at -80 °C until further use.

### Tissue processing, RNA extraction and cDNA synthesis

Frozen tissue samples (approx. 100 mg) were defrosted and homogenized in a Tripure Isolation Reagent in a Roche MagNA Lyser instrument, and RNA phase was separated. The method for RNA isolation was applied according to the manufacturer's instructions. The RNA samples obtained were kept frozen at -80 °C, to be used later. cDNA was synthesized by real-time (RT)-PCR using a Roche Transcriptor High Fidelity cDNA Synthesis Kit.

### Quantitative RT-PCR analysis

cDNA samples were amplified in RT-PCR with a Roche Light Cycler 480 Probes Master Kit using Taq DNA polymerase (Roche; Mannheim, Germany). This method was applied according to the manufacturer’s instructions and specific forward and reverse primers and probes were used for each gene amplification designed by the kit manufacturer. The hTERT, pontin, reptin and dyskerin expression profiles were determined human β-actin was amplified as an reference gene. mRNA expression measurements in tumour and normal tissues were repeated 3 times. Relative quantification (RQ) values were calculated using the formula shown below:

RQ = 2[C_T_(tumour, reference gene) - C_T_(tumour, target gene)] / 2[C_T_ (calibrator, reference gene) - C_T_(calibrator, target gene)]

### Statistical analysis

Statistical analyses were performed using SPSS 22.0 software (IBM Corp., Armonk, NY, USA). The data were presented as means (standard deviation), and statistical analyses were performed using the paired-sample t-test and Wilcoxon signed-rank test. Pearson correlation coefficients were calculated to determine the relationship between the parameters. For all analyses, a p-value <0.05 was regarded as statistically significant.

## RESULTS

### mRNA expressions in all patients

**Figure 1:**
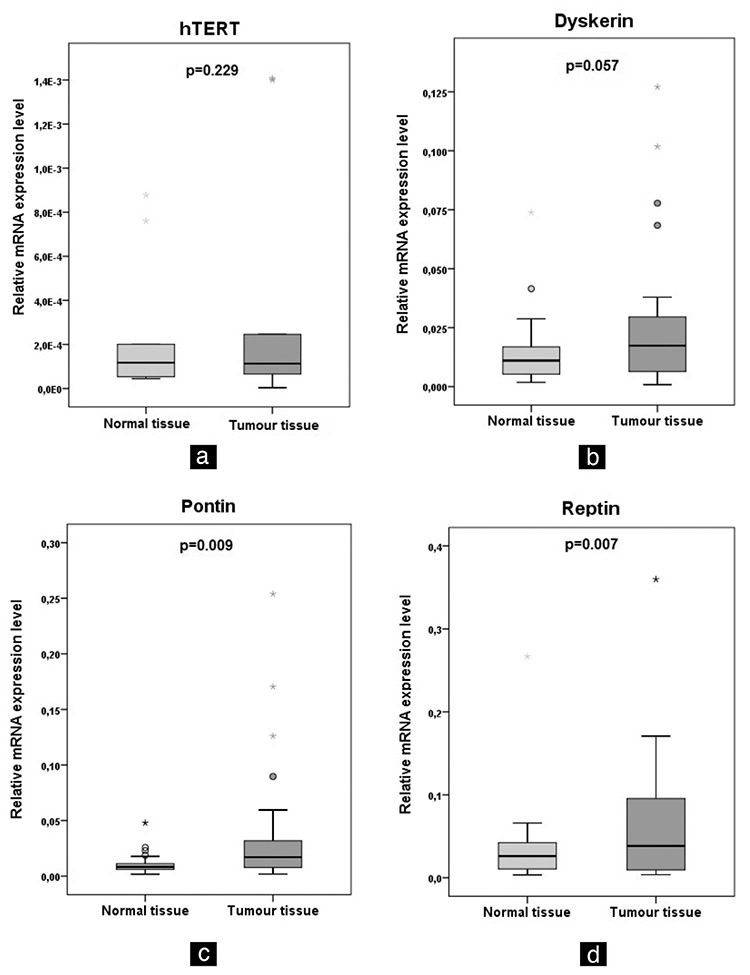
Expression of hTERT (a), dyskerin (b), pontin (c) and reptin (d) in normal and tumour tissues. The hTERT, pontin, reptin and dyskerin mRNA expression in the tumour tissues and corresponding normal tissues of the same patients was evaluated using RT-PCR.

mRNA expressions of hTERT (Figure 1a) and dyskerin (Figure 1b) were higher, but not statistically significant, in tumour tissues than those in the normal tissues of the patients (p>0.05). Pontin (Figure 1c) and reptin (Figure 1d) mRNA expressions were significantly higher in tumour tissues than those in the normal tissues of all the patients (p<0.01). Pontin mRNA expressions in patients with colon cancer and reptin mRNA expressions in patients with breast cancer were significantly higher in tumour tissues than those in normal tissues (p<0.05 and p<0.05, respectively).

In all the patients, 53.8% (14/26) of the samples for hTERT, 73.1% (19/26) of those for pontin, 57.7% (15/26) of those for reptin and 53.8% (14/26) of those for dyskerin showed higher mRNA expression ratios (RQ>1) in tumour tissues than in normal tissues.

### Correlation of hTERT, pontin, reptin and dyskerin expressed in cancer

Three genes, but not hTERT, were expressed in both normal and tumour tissues of all patients. Only ten of the patients showed both tumour and normal tissue expressions of hTERT. In the other 16 patients, 7 showed expression of hTERT in their tumour tissues, but not in normal tissues.

For all patients, there were significantly positive correlations between hTERT, pontin and reptin, in both normal and tumour tissues (Table 2). Dyskerin was positively correlated with pontin and reptin in the normal tissues of all the patients, while it showed significantly high positive correlations with all genes in the tumour tissues (Table 2). When the correlations were investigated according to cancer type, there were significantly positive correlations between hTERT, pontin and reptin in the normal tissues, and between hTERT, pontin, reptin and dyskerin in the tumour tissues of the patients with colon cancer (Table 2). In the breast cancer group, there was a positive correlation between pontin and reptin in the normal and tumour tissues (Table 2). In the gastric cancer group, pontin, reptin and dyskerin were correlated with each other in tumour tissues (Table 2).

## DISCUSSION

It has been shown that telomerase activity is determined frequently in tumour tissues but rarely in normal tissues (11). Most malignant cancers (85-90%) are known to show high telomerase activity for cellular immortality (12). Although the absence of telomerase in normal cells is considered a natural defence against the development of cancer, telomerase is thought to reappear in cancer cells through multiple mechanisms (13). hTERT is the rate-limiting determinant in telomerase activation (14). In the current study, it was found that hTERT mRNA expressions were higher in tumour tissues than in the normal tissues of almost half of the patients. Palmqvist et al. (15) determined a positive telomerase activity in 77%, and varied levels of hTERT mRNA expressions in 91% of cases in their study conducted with colorectal cancer patients. In another study, hTERT expressions were observed in all tumour samples from colorectal cancer patients and found to be significantly higher than those in normal tissues (16). In this study, only one patient had no expression of hTERT in both normal and tumour tissues in the colon cancer group. Elkak et al. (17), in their study conducted with 116 breast tumours and 31 normal breast tissues, reported that hTERT mRNA expressions were observed in all the cancer tissues and in most of the normal tissues, and tumour tissue expressions were approximately 2.5 times higher than those of the normal tissues. In this study, 3 patients with early-stage breast cancer showed hTERT mRNA expressions in tumour tissues, but none in normal tissues.

Additional factors are needed for full activity of the telomerase complex besides TERT and TERC. Dyskerin, which is one of them, directly binds to TERC and stabilizes it in the complex. Loss of the function of dyskerin decreases the steady-state levels of TERC, reduces telomerase activity and causes premature telomere shortening (6). Montanaro et al. (18) showed in their study that dyskerin mRNA levels were quite variable in breast cancer patients and were directly related to TERC. They claimed that decreased dyskerin expression was a sign of a better clinical outcome and prognosis. In this study, mean dyskerin mRNA expressions were higher, but not statistically significant, in tumour tissues than in normal tissues in the breast cancer group.

Pontin and reptin are eukaryotic AAA+ proteins and they participate in different pathways (9). The expressions of these proteins are thought to be increased in many types of cancer (19). Flavin et al. (12) showed positive reptin expression in 60% of colon cancer samples in their study. In this study, mean reptin mRNA expressions were also significantly higher in tumour tissues than in normal tissues in both total and breast cancer patients. Mean pontin mRNA expressions were also significantly higher in tumour tissues than in normal tissues in both total and colon cancer patients.

It is claimed that TERT complexes are dynamic and thus there are at least two different forms of TERT complexes. One of these forms is the pontin/reptin/TERT complex (pretelomerase complex), which has low telomerase catalytic activity. The other one is the TERT/TERC/dyskerin complex (mature telomerase complex), which has high enzymatic activity. In a step-by-step assembly model, a pretelomerase complex is formed, then reshaped to form a mature telomerase complex (1). So, although the TERT/TERC/dyskerin complex in tumour cells is accepted as the leader form, pontin and reptin gene expressions must also be expected to increase. This study was based on investigating components located in this complex in different types of cancer. When correlations of genes were examined against each other, TERT only showed a high correlation with pontin, and no correlation with reptin and dyskerin in normal tissues, both in all patients and the colon cancer group. However, a high positive correlation was observed among all genes in tumour tissues. This can be interpreted as an increase in telomerase activity in tumour cells. Flavin et al. (12) showed a positive correlation between hTERT and reptin in colon cancer. It is thus believed that making an evaluation in terms of hTERT in breast cancer patients would not be correct due to the differences in the results obtained. However, although there were simultaneous increases or decreases in expressions in individual patients with breast cancer, a strong positive correlation could only be seen between pontin and reptin in normal and tumour tissues. Montanaro et al. (18) showed a strong relationship between decreased dyskerin mRNA values and reduced telomerase activity in breast cancer. Although no correlation between hTERT and other genes in gastric cancer patients was found in this study, positive correlations were found between pontin, reptin and dyskerin just in the tumour tissues of the patients. Li et al. (20) showed a positive relationship between reptin and TERT expressions in gastric cancer. The TERT gene is the direct target of reptin and transcription of TERT requires reptin expression and its cooperation with c-Myc. Thus, reptin regulates telomerase at two different levels.

In conclusion, this is the first study to compare hTERT, pontin, reptin and dyskerin expressions in normal and tumour tissues of the same patients. Mostly, increased mRNA expressions, in tumour tissues of all genes, show their role in cancer development. In addition, correlations of pontin, reptin and dyskerin with hTERT support the hypotheses describing their roles in the formation of the telomerase complex in cancer. Furthermore, due to the lack of expression of hTERT in some normal tissues, while being present in tumour tissues, it may be a potentially valuable diagnostic marker of cancer, especially of breast cancer.

## Figures and Tables

**Table 1:**
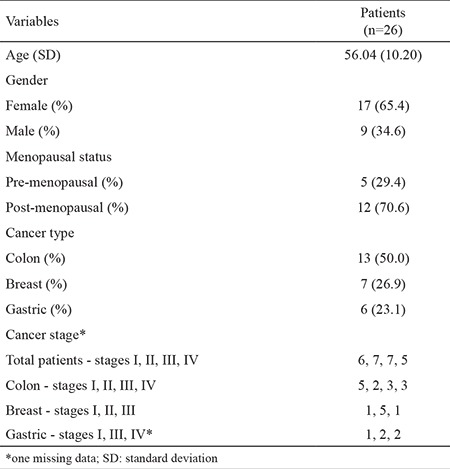
Baseline characteristics of the patients

**Table 2:**
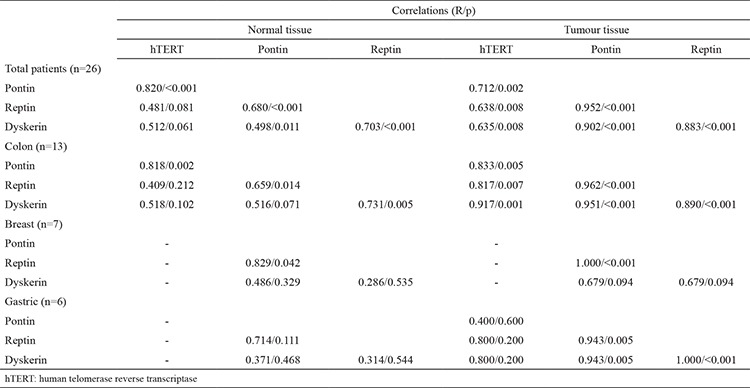
Correlations between mRNA expressions of the genes in both normal and tumour tissues in both overall and classified according to the type of cancer
